# *CEBPA*-associated familial acute myeloid leukemia mimicking Werner syndrome: a case report

**DOI:** 10.3389/fgene.2025.1575651

**Published:** 2025-04-17

**Authors:** Tanguy Demaret, Damien Feret, Barbara Lambert, Delphine Pranger, Jean-Louis Dargent, Maria Dolores Martin Martinez, Antonio Renda, Isabelle Maystadt

**Affiliations:** ^1^ Centre de Génétique Humaine, Institut de Pathologie et Génétique, Gosselies, Belgium; ^2^ Service d’Hématologie, Grand Hôpital de Charleroi (GHdC), Charleroi, Belgium; ^3^ Centre d’Anatomopathologie, Institut de Pathologie et Génétique, Gosselies, Belgium; ^4^ Service d’Urologie, Grand Hôpital de Charleroi (GHdC), Charleroi, Belgium; ^5^ Département de Médecine, Unité de Recherche en Physiologie Moléculaire (URPhyM), Université de Namur (UNamur), Namur, Belgium

**Keywords:** whole exome sequencing, personalized medicine, tumor sequencing, case report, phenocopy

## Abstract

*CEBPA*-associated familial acute myeloid leukemia (AML) is an autosomal dominant leukemia predisposition syndrome associated with germline variants in the *CEBPA* gene. Werner syndrome (WS) is an autosomal recessive progeroid syndrome causing premature aging and malignancies (e.g., AML). We report a 41-year-old man referred for medical genetic evaluation because of 3 synchronous tumors (colon, kidney, and thyroid) and premature aging. He underwent hematopoietic stem cell transplantation (HSCT) at 12 years of age because of AML diagnosed 3 years earlier. His sister (donor for the HSCT) and his brother later developed AML, as did two of her sister’s three children. The patient met the clinical criteria for a “probable” WS, but duo-based (urine and blood DNA) whole exome sequencing did not confirm this diagnosis. A heterozygous c.350del p.(Gly117Alafs*43) pathogenic variant in the *CEBPA* gene was found in the proband’s urine and blood DNA, and in his affected relatives. We postulate that AML management led to adverse effects in the proband, mimicking a WS phenotype (phenocopy). To our knowledge, this is the first report of a leukemia predisposition syndrome mimicking a progeroid syndrome. The diagnosis allowed for personalized medicine (i.e., lifelong regular complete blood count check-up) in the proband and his affected relatives.

## 1 Introduction

*CEBPA*-associated familial acute myeloid leukemia (AML) is an autosomal dominant predisposition to AML confered by germline (likely) pathogenic variant affecting the N-terminal extremity of the protein (upstream of the codon 120) (Smith et al., 2004; Tawana and Fitzgibbon, 1993). The *CEBPA* gene encodes the CCAAT/enhancer-binding protein alpha, a transcription factor crucial for myeloid differentiation (Avellino and Delwel, 2017). *CEBPA* mRNA has two translation initiation sites at codons 1 and 120, resulting in the synthesis of a full-length (42 kDa, p42) and a shorter (30 kDa, p30) isoform of C/EBPα, respectively. p42 and p30 homo- and hetero-dimerize, and their ratio influences cell fate: p42 inhibits cell proliferation and promotes differentiation, whereas p30 preponderance is associated with an immature cell state and inhibition of terminal differentiation (Schmidt et al., 2020). The proposed leukemogenesis mechanism is based on a “second hit” model (Knudson, 1971): the germline variant frequently affects the p42 isoform (the “cell differentiation” isoform, upstream of codon 120) while the somatic variant (downstream of codon 120) affects both isoforms, leaving intact only one copy of p30 (the “anti-cell differentiation” isoform) on the allele affected by the germline variant. The final consequence is thought to be a gain-of-function mechanism induced by a dramatically decreased p42/p30 ratio (Schmidt et al., 2020). *CEBPA*-associated familial AML is a highly penetrant predisposition with favorable long-term outcomes (Pan et al., 2024; Tawana et al., 2015). Carrier surveillance is based on complete blood count (CBC) every 6–12 months, followed by bone marrow examination in case of CBC anomalies.

Werner syndrome (WS) is an autosomal recessive progeroid syndrome caused by germline bi-allelic (likely) pathogenic variants in *RECQL2,* encoding the WRN protein, which is important for DNA repair and telomere maintenance (Rossi et al., 2010). Patients develop normally (until adolescence) before presenting features of accelerated aging: skin atrophy, wrinkles, bilateral cataracts, hypogonadism, early loss of fertility, short stature, high-pitched hoarse voice, osteoporosis, type 2 diabetes, and indolent ulceration around the Achilles tendon (Oshima et al., 2017). Early death (around 60 years) mostly results from myocardial infarction (secondary to premature atherosclerosis) or malignancies (leukemia, sarcoma, thyroid follicular carcinomas, - among others).

For the first time, we report a patient presenting with a possible clinical diagnosis of WS (bilateral cataracts, premature aging, short stature, hypogonadism, and multiple malignancies) who was finally diagnosed with *CEBPA*-associated familial AML through whole exome sequencing. This diagnosis allowed for the implementation of personalized medicine strategies.

## 2 Case description

Aged 41, the proband presented with 3 synchronous tumors, detected by a full-body positron emission tomography and computed tomography (PET-CT) performed during the work-up of anemia revealed by pallor and fatigue. A tubulovillous adenoma (high-grade dysplasia) of the right colic flexure, associated with multiple colon polyps (low-grade dysplasia), and an unclassified renal cell carcinoma were resected by right colectomy and partial right nephrectomy, respectively. Additionally, the patient declined the resection of a hypermetabolic nodule located inside a nodular goiter, also detected during PET-CT.

**FIGURE 1:**
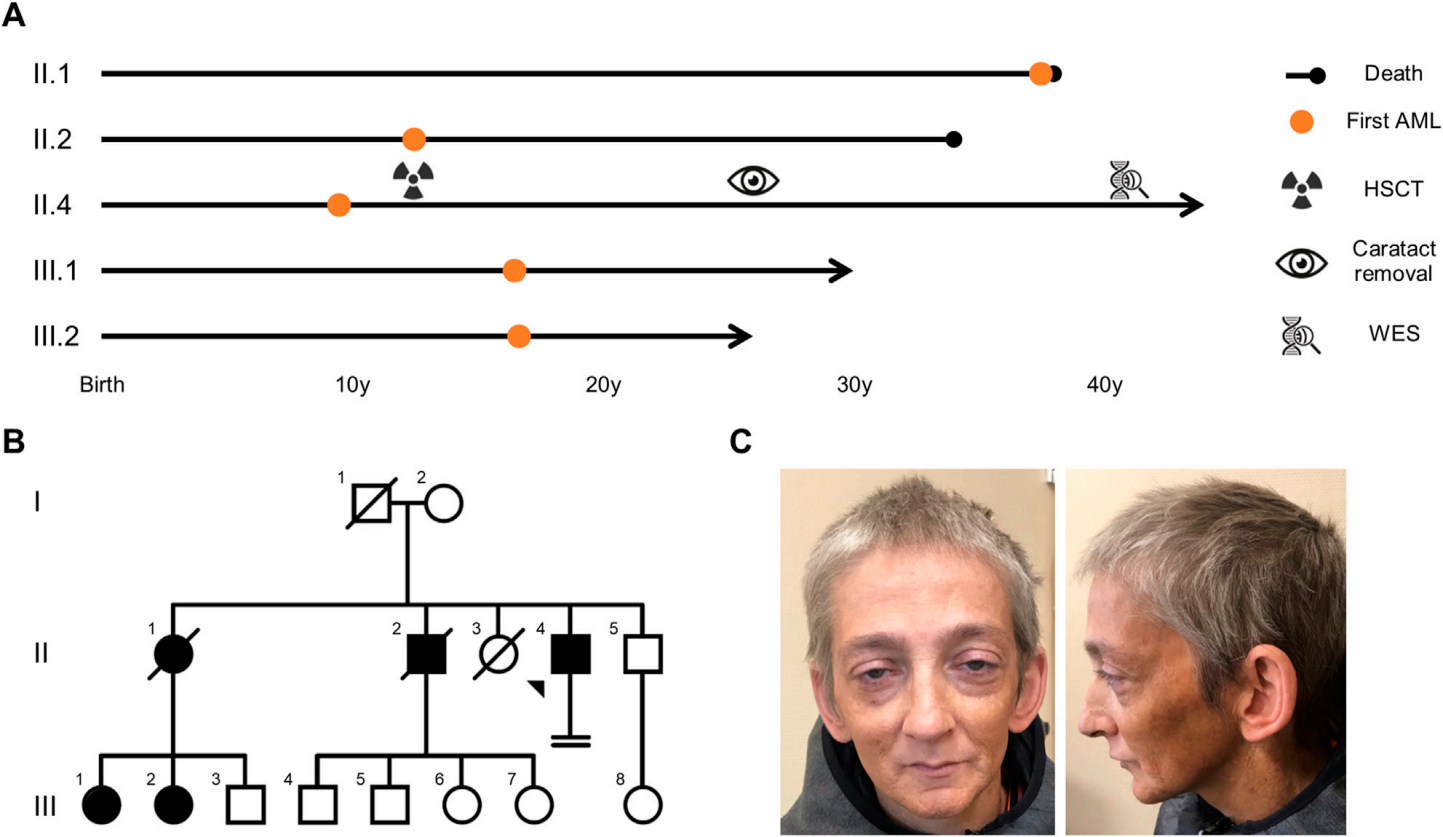
AML segregated over two generations in the proband’s pedigree, with an age of onset comprised between 9 and 37 years of age. **(A)** Overview of the proband’s and his relatives’ medical journey. **(B)** Family tree presenting females (circles) and males (squares) affected (filled symbols) by AML. Arrow: proband, crossed symbol: deceased, horizontal double lines: infertility. **(C)** Proband’s face and profile photographs (taken at 41 years) highlighting premature aging and bilateral ptosis. AML: acute myeloid leukemia, HSCT: hematopoietic stem cell transplantation, WES: whole exome sequencing.

His medical history was marked by an AML, at the age of 9, managed with chemotherapy (Figure 1A). Three years later, at 12 years, he received hematopoietic stem cell transplantation (HSCT, from his older sister) to manage disease recurrence. The HSCT was pre-conditioned by total body irradiation and was complicated by a graft-versus-host-disease (GVHD), which subsequently resolved. Complete donor chimerism was confirmed on a bone marrow sample at 28 years. He further developed bilateral cataracts (surgically corrected at 26 years), hypergonadotropic hypogonadism (intermittently treated with intramuscular injections of androgens), pulmonary emphysema (in the context of tobacco product exposure), osteoporosis, hepatic steatosis, hypercholesterolemia, hypertriglyceridemia, and depression.

He is the fourth child of unrelated parents originating from Belgium with no known hematological malignancies in their ancestors. Their two eldest children died of AML at the ages of 38 years (AML diagnosed at 37) and 34 years (AML diagnosed at 12), respectively (Figure 1B). The oldest of the two was the donor for the proband’s HSCT. She had 3 children, two of whom developed AML before the age of 18.

Clinical examination showed a progeroid appearance (Figure 1C) characterized by premature graying of hair, bilateral ptosis, a beaked nose, wrinkles, a prematurely aged face, and short stature (156.5 cm and 35 kg, corresponding to a body-mass index of 14.3 kg/m^2^). The patient also presented with a hoarse high-pitched voice.

## 3 Diagnostic assessment

Based on the multiple tumors, the proband’s urologist requested the sequencing of a gene panel including 38 genes (Supplementary Table S1) involved in hereditary cancer syndromes (Roche KapaHypercapV3 capture followed by Illumina sequencing, mean coverage 3582x). Testing was performed on DNA extracted from blood (patient’s sister DNA), urine, and buccal swab (patient’s own DNA). The analysis revealed no (likely) pathogenic variant explaining the proband’s phenotype (*WRN* and *CEBPA* genes were not included, see below).

**FIGURE 2:**
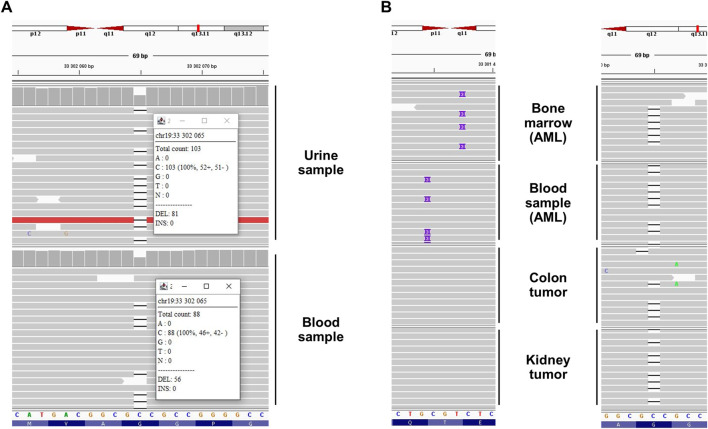
Next-generation sequencing results highlighted a germline familial c.350del *CEBPA* variant related to *CEBPA*-associated familial AML in **(A)** the proband’s urine (own DNA) and blood (sister’s DNA, due to HSCT) and **(B)** the proband’s (colon and kidney tumors) and relatives’ (bone marrow and blood) samples. In the bone marrow and blood samples, somatic variants (c.925_927dup and c.930_931insAAG, respectively) affecting the C-terminal extremity of the protein were detected (“second hit”) in addition to the germline variant. Integrative Genomics Viewer screenshots with black dash and purple rectangle representing deletion of one nucleotide and insertion of three nucleotides, respectively. Note that *CEBPA* is located on the minus strand of the chromosome. Thus the 5′ extremity is presented on the right side of the figure. AML: acute myeloid leukemia, HSCT: hematopoietic stem cell transplantation.

The patient was referred to our center for further medical genetic evaluation. Based on the patient’s phenotype, the hypothesis of WS was considered. The patient met the clinical criteria for a “probable” WS (Table 1 (Oshima et al., 2017)). A duo-based (DNA isolated from urine and blood, the latter being the patient’s sister DNA) whole exome sequencing (Roche KAPA HyperExome capture followed by Illumina sequencing, mean coverage 300x) revealed a heterozygous c.350del p.(Gly117Alafs*43) variant in the *CEBPA* gene (NM_004364.5) (Figure 2) which was confirmed by Sanger sequencing (ClinVar SCV005387846.1). The variant was not found in large databases (gnomAD and ClinVar) or in our in-house exome database. It affects the N-terminal end of the protein and segregates with the disease (see below). According to the American College of Medical Genetics criteria, this variant was classified as pathogenic (class 5: PVS1, PS3, PM1, PM2, and PP1) (Richards et al., 2015). No class 3 variant (or higher) was detected in the *WRN* gene, thus providing no molecular evidence for WS to explain the patient’s phenotype (phenocopy).

**TABLE 1:** Werner syndrome diagnostic criteria (Oshima et al., 2017).

	Proband
Cardinal signs and symptoms (onset over 10 years old)	
Bilateral cataracts	+
Characteristic dermatological pathology and facies^a^	+
Short stature	+
Consanguinity^b^ or affected sibling	
Premature greying and/or thinning of scalp hair	+
Further signs and symptoms	
Diabetes mellitus	
Hypogonadism^c^	+
Osteoporosis	+
Osteosclerosis of distal phalanges of fingers and/or toes^d^	
Soft tissue calcification	
Evidence of premature atherosclerosis^e^	
Mesenchymal/rare/multiple neoplasms	+
Voice changes^f^	+
Flat feet	

Proband’s signs and symptoms matched the criteria for “probable diagnosis” of Werner syndrome. Definite: all the cardinal signs and two further signs, probable: the first three cardinal signs and any two others, possible: either cataracts or dermatological alterations and any four others. Exclusion criteria: onset of signs and symptoms before adolescence (except stature).

^a^ Consisting of tight skin, atrophic skin, pigmentary alterations, ulceration, hyperkeratosis, regional subcutaneous atrophy and “bird” facies.

^b^ Third cousin or greater.

^c^ Consisting of secondary sexual underdevelopment, diminished fertility, testicular or ovarian atrophy.

^d^ X-ray diagnosis.

^e^ History of myocardial infarction.

^f^ High-pitched, squeaky, or hoarse voice.

+: present.

The molecular mechanism leading to AML associated with a germline *CEBPA* variant is based on a somatic second hit at the C-terminal extremity of the protein (after codon 120) (Pabst et al., 2008; Pabst and Mueller, 2009). This mechanism was confirmed in blood and bone marrow samples from two relatives (Individuals II.2 and III.1) during AML (Table 2, Figure 2). We hypothesized that this mechanism could also underlie the tumorigenesis of the colon adenoma and the unclassified renal cell carcinoma. However, both tumor DNA sequencing analyses (Archer VARIANTPlex Core Myeloid amplification followed by Illumina sequencing, mean *CEBPA* coverage 2080x and 3560x) detected no second variant in the *CEBPA* gene (Table 2, Figure 2), and could not support our hypothesis for solid tumorigenesis in the proband. Tumor DNA methylation profiling array (Infinium MethylationEPIC BeadChip from Illumina) detected partial chromosomal losses and amplifications (Supplementary Figures S1, 2) but failed to provide information about tumor classification and/or tumorigenesis mechanisms. *MGMT* promoter was not found to be methylated (Bady et al., 2016).

**TABLE 2:** *CEBPA* sequencing results.

	I.2	II.1	II.2	II.4 (proband)	II.5	III.1
Samples						
Blood	c.[=];[=]	c.[350del];[=]^a^	c.[350del];[930_931insAAG]^b^	c.[350del];[=]^c^	c.[=];[=]	
Bone marrow						c.[350del];[925_927dup]^4^
Urine	c.[=];[=]			c.[350del];[=]		
Buccal swab	c.[=];[=]					
Colic tumor				c.[350del];[=]		
Kidney tumor				c.[350del];[=]		
Interpretation						
Germline variant	Not carrier	Carrier	Carrier	Carrier	Not carrier	Carrier
Somatic variant	n.a.	No sample available for somatic analysis	Second hit found in AML blood sample	No second hit in sampled solid tumors	n.a.	Second hit in AML bone marrow

*CEBPA* (NM_004364.5) variants detected in various samples collected from the pedigree confirmed: (1) the carrier status of relatives affected by acute myeloid leukemia (AML), and (2) the leukemogenesis mechanism (i.e., a molecular second hit in *CEBPA* gene, in AML samples). Urine and buccal swab were collected in I.2 to exclude somatic mosaicism of the familial (i.e., germline) *CEBPA*, variant.

^a^ Sequenced in the proband who received HSCT, from II.1.

^b^ Blood was sampled during AML. Somatic allele consequence is p.(Thr310_Gln311insLys) and allelic frequency was 0.24.

^c^ Corresponds to the genome of his sister (II.1 is the donor for the proband’s HSCT).

^d^ Bone marrow was sampled during AML. Somatic allele consequence is p.(Glu309dup) and allelic frequency was 0.42.

n.a.: not applicable, AML: acute myeloid leukemia, HSCT: hematopoietic stem cell transplantation.

Maternal follow-up testing in three different samples (blood, urine, and buccal swab) did not detect the familial *CEBPA* variant. The father could not be tested (deceased from a non-malignant cause at the age of 66) and there was no history of hematological malignancy on his side of the family. The recurrence of the pathogenic variant in 3 out of 5 siblings suggests the hypothesis of parental germline mosaicism as a potential explanation, especially considering that maternal screening was negative and both the father’s personal medical history and family history were unremarkable for hematological malignancies.

## 4 Discussion

We report an adult patient presenting with three synchronous tumors (colon, kidney, and thyroid) following childhood (ages 9–12) AML management with chemotherapy, total body irradiation, and HSCT received from his deceased sister (who died of AML at 38 years). WS was clinically suspected but not confirmed by WES which revealed a pathogenic (class 5) truncating variant in the *CEBPA* gene, associated with familial AML, in the patient’s urine (own DNA) and blood (sister’s DNA). Blood and bone marrow sequencing confirmed the leukemogenesis mechanism by the presence of a second hit in the *CEBPA* gene in two relatives affected by AML. No molecular second hit was found in the two solid tumors resected from the proband, thus making the tumorigenesis mechanism unlikely related to the germline *CEBPA* variant.

The germline origin of the c.350del p.(Gly117Alafs*43) *CEBPA* variant has been confirmed on three different samples collected from the proband (urine, kidney and colon) and on three relatives’ blood/bone marrow samples (i.e., familial inheritance of the variant). These results allow to firmly confirm the germline origin of the variant without requiring skin fibroblast DNA sequencing [i.e., the gold-standard method to confirm germline origin (Desai et al., 2017)].

The occurrence of a somatic variant in the *CEBPA* gene, known as the “second hit”, is the mechanism leading to AML in patients carrying a germline *CEBPA* variant, as confirmed in this pedigree. However, we found no evidence to support the same mechanism explaining the colon and kidney tumorigenesis in the patient. Interestingly, a 44-year-old patient carrying a germline *CEBPA* variant died of rectal cancer, without prior AML treatment (Pathak et al., 2016). This case suggests a potential predisposition to solid tumor associated with germinal *CEBPA* variants. Although this association has not been confirmed, it warrants further evaluation in future studies, given that CEBPA downregulation has been observed in solid tumors and is a potential therapeutic target (Chan et al., 2019; Koschmieder et al., 2008).

The proband presented with several signs and symptoms reported in WS (Table 1), yet no molecular etiology could be found to support this diagnosis. It is likely that all the harbored WS criteria are late effects of the leukemia management (alkylating agents and corticosteroid leading to cataracts, TBI causing dyslipidemia, gonadal dysfunction, and second cancers) (Chow et al., 2016; Inamoto and Lee, 2017) or their consequences (dermatological pathology can be secondary to GVHD, and hypogonadism can explain the short stature, premature hair graying and progeroid appearance) (Phelan et al., 2022).

Xiao and colleagues reported a 36-year-old patient who presented with donor cell leukemia (DCL) 13 months after having received HSCT (from his healthy sister) to manage AML (Xiao et al., 2011). *CEBPA* gene sequencing revealed the presence of a germline variant in the patient and the donor (his sister), meaning that both were predisposed to AML. In our case, the proband faces the same risk as the patient reported in this case report, because both have received HSCT from a donor carrying a germline *CEBPA* variant. Consequently, these findings suggest that CEBPA gene screening should be considered prior to any intrafamilial HSCT in the context of AML, to prevent the use of donors carrying germline CEBPA variants.

One of the limitations of this study is that we were not able to obtain precise information about the initial AML management in the proband (dose and duration of chemotherapy, dose of irradiation, etc.), which impeded drawing a precise correlation between the intensity of the management and the adverse effects presented by the patient. In addition, we had no access to the proband’s AML sample, which precluded establishing an unequivocal link between the AML of the proband and the *CEBPA* gene AML predisposition. However, this link has been confirmed in two relatives (individuals II.2 and III.1, Table 2). Finally, the fact that we had no thyroid tumor sample, and that WES was not performed on all samples (due to funding limitations), is restraining further the deciphering of tumorigenesis mechanisms.

Thanks to WES, we were able to identify the etiology of AML segregating in a pedigree, which allowed for personalized medicine. Further genetic testing (i.e., tumor *CEBPA* sequencing) provided no evidence supporting a link between the solid tumors presented by the proband and the AML predisposition. The diagnosis provided a recurrence risk of AML and a personalized surveillance plan for the proband and his relatives. It will also prevent using a *CEBPA* variant carrier as familial donor for HSCT in the future.

## 5 Patient perspective

The genetic diagnosis was able to exclude the severe diagnosis of WS in the proband. Yet, the AML treatment by HSCT received from his sister who later developed and died of AML, made him aware that he was at risk for DCL well before the genetic diagnosis was known. He told us that the genetic diagnosis (i.e., CEBPA-associated familial AML) was a formal confirmation of what he had already guessed some years ago.

The proband has been advised to do a check-up with CBC every 6 months.

## Data Availability

The raw data supporting the conclusions of this article will be made available by the authors, without undue reservation.
