# Supplementary material for: CEBPA-associated familial acute myeloid leukemia mimicking Werner syndrome: a case report
Source: Front Genet. 2025 Apr 17;16:1575651. doi: 10.3389/fgene.2025.1575651 (PMC12043886; doi:10.3389/fgene.2025.1575651)
Supplement: Supplementary file 1 [file DataSheet1.docx]

Supplementary Material

# Supplementary Table

| *APC* | *CDKN2A* | *MUTYH* | *RAD51C* |
| --- | --- | --- | --- |
| *ATM* | *CHEK2* | *NBN* | *RAD51D* |
| *BAP1* | *EPCAM* | *NTHL1* | *RPS20* |
| *BARD1* | *GATA2* | *PALB2* | *SCG5* |
| *BMPR1A* | *GREM1* | *PIK3CA* | *SMAD4* |
| *BRCA1* | *MEN1* | *PMS2* | *STK11* |
| *BRCA2* | *MLH1* | *POLD1* | *TP53* |
| *BRIP1* | *MSH2* | *POLE* | *WWP1* |
| *CDH1* | *MSH3* | *PTEN* |  |
| *CDKN1B* | *MSH6* | *RAD50* |  |

**Supplementary Table 1. List of the 38 genes included in the “hereditary cancer syndromes” gene panel.**

# Supplementary Figures

**Supplementary Figure 1.** **Colon tumor DNA methylation profiling array showed 1p and 18q deletion.** Chromosome 1 to 22 amplifications and losses represented as positive and negative deviations from baseline, respectively.


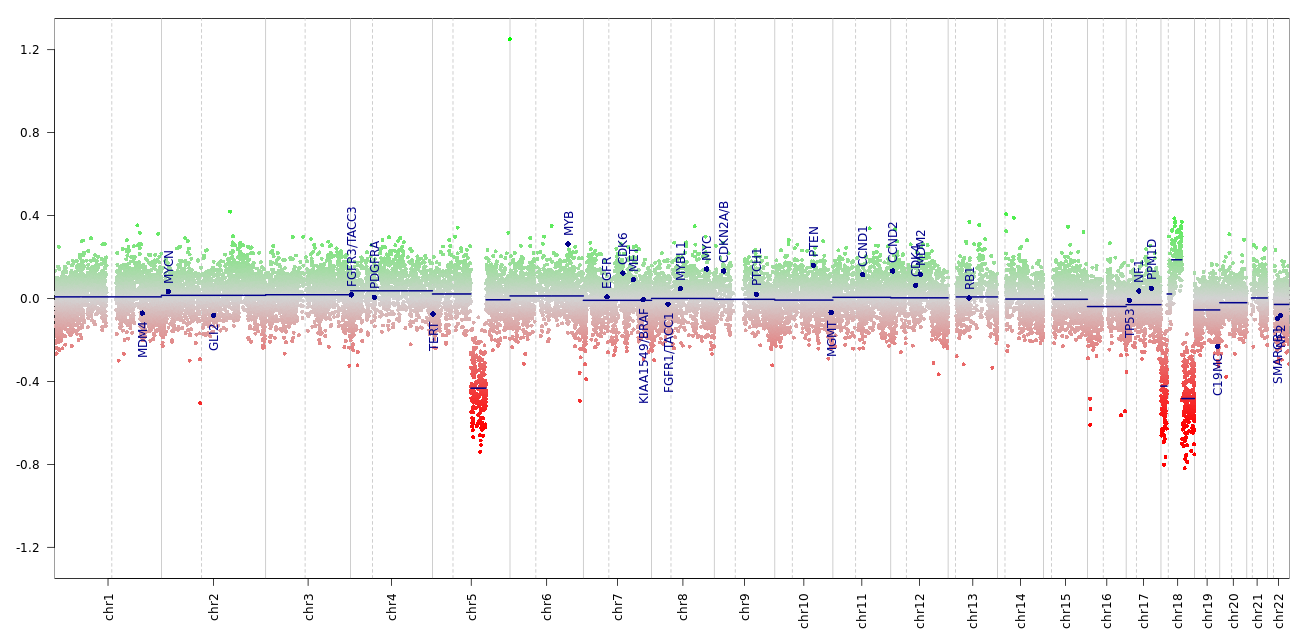


**Supplementary Figure 2.** **Renal tumor DNA methylation profiling array showed partial 5p, 18p and 18q deletion, and partial 18q duplication.** Chromosome 1 to 22 amplifications and losses represented as positive and negative deviations from baseline, respectively.
